# The influence of alcohol on genioglossus single motor units in men and women during wakefulness

**DOI:** 10.1113/EP090580

**Published:** 2022-12-19

**Authors:** Joanne Avraam, Andrew Dawson, Christian L. Nicholas, Monika D. Fridgant, Feiven Lee Fan, Amanda Kay, Zi Yi Koay, Rachel Greig, Fergal J. O'Donoghue, John Trinder, Amy S. Jordan

**Affiliations:** ^1^ Melbourne School of Psychological Sciences University of Melbourne Melbourne Australia; ^2^ Department of Respiratory and Sleep Medicine and Institute for Breathing and Sleep Austin Health Heidelberg Victoria Australia; ^3^ Faculty of Medicine University of Melbourne Parkville Victoria Australia

**Keywords:** ethanol, obstructive sleep apnoea, pharyngeal muscles, snoring, upper airway

## Abstract

Alcohol worsens obstructive sleep apnoea (OSA). This effect is thought to be due to alcohol's depressant effect on upper airway dilator muscles such as the genioglossus, but how alcohol reduces genioglossal activity is unknown. The aim of this study was to investigate the effect of alcohol consumption on genioglossus muscle single motor units (MUs). Sixteen healthy individuals were studied on two occasions (alcohol: breath alcohol concentration ∼0.07% and placebo). They were instrumented with a nasal mask, four intramuscular genioglossal EMG electrodes, and an ear oximeter. They were exposed to 8–12 hypoxia trials (45–60 s of 10% O_2_ followed by one breath of 100% O_2_) while awake. MUs were sorted according to their firing patterns and quantified during baseline, hypoxia and recovery. For the alcohol and placebo conditions, global muscle activity (mean ± SD peak inspiratory EMG = 119.3 ± 44.1 and 126.5 ± 51.9 μV, respectively, *P* = 0.53) and total number of MUs recorded at baseline (68 and 67, respectively) were similar. Likewise, the peak discharge frequency did not differ between conditions (21.2 ± 4.28 vs. 22.4 ± 4.08 Hz, *P* = 0.09). There was no difference between conditions in the number (101 vs. 88, respectively) and distribution of MU classes during hypoxia, and afterdischarge duration was also similar. In this study, alcohol had a very minor effect on genioglossal activity and afterdischarge in these otherwise healthy young individuals studied while awake. If similar effects are observed during sleep, it would suggest that the worsening of OSA following alcohol may be related to increased upper airway resistance/nasal congestion or arousal threshold changes.

## Abstract (highlights)

- **What is the central question of this study?**
  How does alcohol intake, which worsens obstructive sleep apnoea, alter motor control of the genioglossus muscle, an upper airway dilator, in healthy awake human volunteers, and does alcohol alter genioglossus muscle afterdischarge?
- **What is the main finding and its importance?**
  Alcohol consumption had a very minor effect on the activity of the genioglossus in healthy young individuals studied during wakefulness and did not alter afterdischarge, leaving open the possibility that alcohol worsens obstructive sleep apnoea via other mechanisms.

## 1 INTRODUCTION

For nearly 40 years it has been reported that alcohol worsens snoring and obstructive sleep apnoea (OSA) (Issa & Sullivan, 1982; Taasan et al., 1981). Recently, two meta‐analyses have outlined the polysomnographic changes that occur when the sleeper is intoxicated (Burgos‐Sanchez et al., 2020; Kolla et al., 2018). Both found modest but statistically significant increases in the severity of OSA (Apnoea–Hypopnoea Index, AHI) of 2–4 events/h. The effect of alcohol was more pronounced in snorers and patients with OSA who experienced 4.2 and 7.1 events/h increase, respectively (Kolla et al., 2018). The mechanism by which alcohol worsens OSA is incompletely understood but warrants investigation given alcohol's widespread use. Reduced genioglossus (GG) muscle activity in response to alcohol is one proposed mechanism that is supported by studies in animals (Bonora et al., 1984; Vecchio et al., 2010) and two studies conducted in awake humans (Krol et al., 1984; Leiter et al., 1987).

**FIGURE 1:**
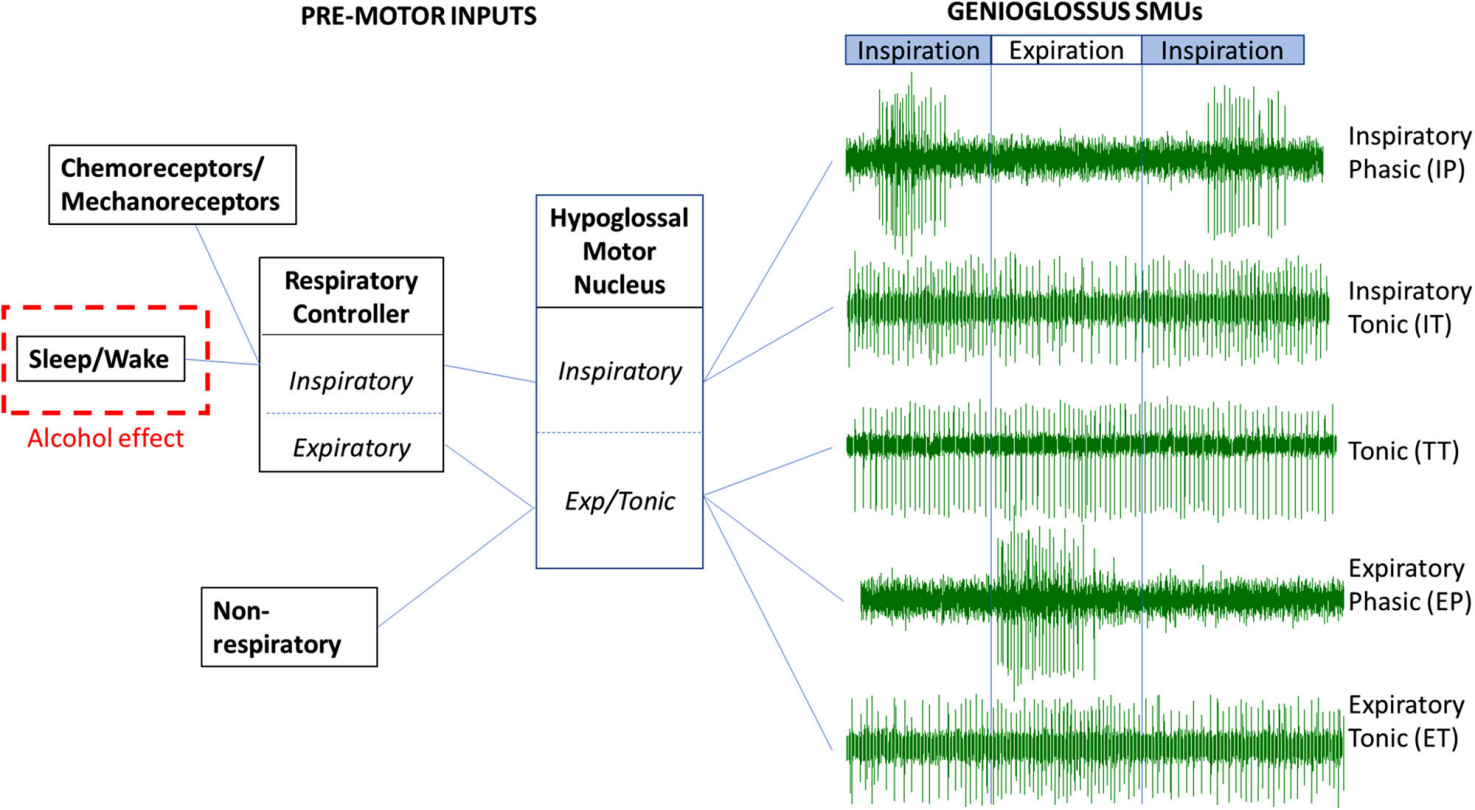
A conceptual model of genioglossus motor control including the hypothesized site of action of alcohol. The five most commonly identified genioglossus MUs are shown on the right, with proposed pre‐motor inputs on the left. The model has arisen from research showing that sleep onset, arousal, CO_2_ and inspiratory resistive loading all principally influence IP and IT units whereas changes to TT/ET and EP units with these stimuli are minimal or not observed (Nicholas et al., 2010; Vecchio et al., 2010; Wilkinson et al., 2008, 2010; Woods et al., 2015). Transition of units between IP and IT units is common (Nicholas et al., 2010; Wilkinson et al., 2010), and likewise conversion of EP/ET to TT and vice versa have been observed (Nicholas et al., 2010). However, transitions between IP/IT and EP/ET/TT firing patterns are very rare suggesting the IP/IT and EP/ET/TT systems are largely distinct.

We, and others, have investigated the motor control of the genioglossus over the past decade (Trinder et al., 2014). These studies have shown that resting genioglossal activity in supine subjects is generated by a combination of five main motor unit (MU) classes each with unique firing patterns (Saboisky et al., 2006). Approximately 30% of genioglossus MUs fire only during inspiration (inspiratory phasic, IP), whereas another 25% fire throughout the respiratory cycle but with increased frequency during inspiration (inspiratory tonic, IT). Approximately 5% of genioglossus MUs fire only during expiration (expiratory phasic, EP) and roughly 15% of MUs fire throughout the breath but with increased frequency during expiration (expiratory tonic, ET). A further 25% of units fire constantly throughout the respiratory cycle (tonic, TT) (Trinder et al., 2014). Importantly, factors that alter genioglossal activity do not alter all unit classes equally, with sleep specifically reducing IP and IT units (Wilkinson et al., 2008), and respiratory stimuli such as loading, hypoxia and hypercapnia also predominantly increasing IP and IT units (Nicholas et al., 2010; Woods et al., 2015). Further, changes in firing patterns between IT and IP patterns are common, and changes between TT/ET and EP have also been observed. However, changes from IP/IT to EP/ET/TT patterns have only very rarely been observed (Nicholas et al., 2010; Walls et al., 2013; Wilkinson et al., 2008, 2010). These observations have led us to develop a two‐compartment conceptual model of the control of the genioglossus (Trinder et al., 2014) (Figure 1) whereby the hypoglossal motor nucleus (HGMN) is composed of two relatively discreet components: an inspiratory system that receives input from the respiratory pattern generator and sleep–wake centres of the brain and outputs to MUs with an inspiratory pattern, and an expiratory/tonic system that receives input from both non‐respiratory centres and an expiratory respiratory pattern generator input, and outputs to expiratory and tonic genioglossus MUs. Which of these systems, or genioglossus MU classes, are altered by alcohol in humans is unknown.

Vecchio and colleagues provided evidence that the effect of alcohol on genioglossal activity in rats is mediated by alcohol's effect on the wakefulness input to the HGMN, rather than direct effects on the HGMN itself (Vecchio et al., 2010). If the effect of alcohol on genioglossus motor control is similar in humans, we would postulate that alcohol would reduce genioglossal activity via a reduction in the activity of IP/IT motor units (MUs), while leaving EP/ET/TT units relatively unchanged (Figure 1). The aim of this study was therefore to assess the influence of alcohol on genioglossus MUs in awake humans. A secondary aim was to investigate the effect of alcohol on the genioglossus afterdischarge (the slow decline in activity after sudden removal of an excitatory stimulus), which we have recently shown to occur predominantly due to IP and IT units (Avraam, Dawson et al., 2021).

## 2 METHODS

### 2.1 Ethical approval

This study was conducted according to the *Declaration of Helsinki* (2013), except for registration in a database, and was granted ethical clearance by the Ethical Committee of The University of Melbourne (ID 11273). All participants provided written informed consent prior to participation.

### 2.2 Participants

Sixteen healthy participants (7 men, 9 women) were studied on two experimental days (placebo and alcohol), 1 week apart. Participants were not informed as to which experimental session was which, but most were able to correctly identify the alcohol from placebo session. Data from the placebo session in these participants have been reported in a recent publication (Avraam et al., 2021). Participants were between 19 and 35 years old, had regular sleep patterns (waking regularly between 07.00 and 10.00 h) with good self‐reported sleep quality and did not smoke or take medications (except the oral contraceptive pill). They had not travelled across time zones or participated in shift work within the previous month. No participant had difficulty breathing through their nose.

### 2.3 Protocol

Participants attended the John Trinder Sleep Laboratory at the University of Melbourne in the morning after breakfast, having refrained from consuming caffeinated drinks or alcohol for the preceding 18 h. Following arrival, the participants’ weight and body water content were measured on bathroom scales and height was measured in order to calculate the alcohol dosage required to produce a breath alcohol concentration (BrAC) of 0.08% for the alcohol condition. Before beginning, the participants were breathalysed (AlcoSense Precision, Andatech Vermont, VIC, Australia) to ensure a baseline BrAC of 0.00%. A glass of Sprite (∼290 ml, equal volume between sessions) containing the alcohol dose (alcohol condition, ∼100 ml of 40% vodka equivalent to about 4.3 UK standard drinks) or with a straw dipped in alcohol (placebo session) was consumed evenly over 30 mins during instrumentation with polysomnography equipment (detailed in 2.4.1 Polysomnography). Thereafter the participant was asked to lie supine on a bed, EMG electrodes were inserted into the genioglossus, and respiratory equipment (detailed below) was fitted.

Once participants were relaxed and comfortable, a practice hypoxia trial was performed to familiarize the participant and to obtain an impression of their hypoxic ventilatory response. After this practice trial and at least 3 min of recovery, resting breathing was recorded for 5 min. Subsequently, 8–12 brief periods of isocapnic hypoxia were delivered to assess afterdischarge as per our prior publication (Avraam et al., 2021). Briefly, each hypoxia trial consisted of a further minute of baseline recording (Baseline) before 45–60 s of hypoxia (Hypoxia – 10% O_2_ in N_2_), which was introduced without warning. Hypoxia was terminated abruptly with one breath of 100% O_2_ (Hyperoxia) and followed by 1 min of room air (Normoxia). Isocapnia was maintained throughout hypoxia and recovery via a manual bleed directly into the mask. Each hypoxic trial was separated by at least 8 min of room air breathing to avoid effects of sustained hypoxia. For some participants with low hypoxic responses, the hypoxia breaths were preceded by up to four breaths of anoxic gas (100% N_2_). This was performed in participants whose ventilation and genioglossal activity increased minimally on the practice or initial trials. BrAC was measured every 20–30 min during room air breathing between hypoxia trials. Upon completion of the protocol, all monitoring equipment was removed and the participant was questioned as to whether they thought it was the alcohol or placebo session if they had not already volunteered this information. They then relaxed in the laboratory until their BrAC reached 0.00% at which point they were allowed to leave.

### 2.4 Instrumentation

#### 2.4.1 Polysomnography

Two EEGs (C3 and O1 referenced to A2), left and right EOG which were referenced to the forehead, and masseter EMG electrodes were recorded to ensure participants remained awake throughout the experiment. Two ECG electrodes were attached to the right clavicle and the left lower ribs to monitor heart rate, and arterial oxygen saturation ($S_{pO_{2}}$) was measured with a pulse oximeter probe (Radical 7 Oximeter, Masimo, Irvine, CA, USA; or Oximax N‐595, Nellcor, Tyco Healthcare, Pleasanton, CA, USA) attached to the right earlobe.

#### 2.4.2 Ventilation

Participants were fitted with a leak‐proof nasal mask (Modified Profile‐Lite, Phillips, Respironics, Murrysville, PA, USA), with heated pneumotachograph (model 3700; Hans Rudolph, Shawnee, KS, USA) attached. The pneumotachograph was connected to a two‐way non‐rebreathing valve (model 2600; Hans Rudolph) that separated inspiratory and expiratory flow, with the inspiratory side of the valve attached to a length of tube which passed through the wall into the control room. In the control room, the tubing was connected to a four‐way tap (model 2600; Hans Rudolph) to allow delivery of the hypoxic, hyperoxic and anoxic gases which were stored in bags (40 and 9 litres, Medisoft, Dinant, Belgium). Mask CO_2_ and O_2_ were continuously sampled from a port in the nasal mask (CD‐3A analyser; Ametek, Berwyn, PA, USA). Normocapnia was maintained throughout the hypoxic gas exposure by administering CO_2_ through a short length of tubing that was connected to the inspiratory side of the non‐rebreathing valve. Mask pressure was monitored (DP45; Validyne, Northridge, CA, USA) through a port in the nasal mask.

#### 2.4.3 Genioglossus muscle activity

Genioglossal activity was recorded using four monopolar intramuscular wire electrodes in accordance with our prior MU publications (Avraam et al., 2021; Nicholas et al., 2010). Each wire was inserted percutaneously via a 25‐gauge hypodermic needle after numbing the submental area for 20–30 min with surface anaesthesia (lidocaine – Prilocaine; AstraZeneca Pty Ltd, North Ryde, NSW, Australia). Wires were inserted between 10 and 20 mm from the posteroinferior margin of the mandible, at a depth of 2–2.5 cm from the surface and ∼5 mm on each side of the midline. The variation in wire placements was performed to maximize chances of identifying unique MUs and to ensure that sufficient EP and ET units were recorded as these are typically found more superficially in the horizontal region of the genioglossus (Luu et al., 2018; Yeung et al., 2022). GG EMG signals were amplified and band‐pass filtered from 30 to 10 kHz (model P511, Grass TeleFactor; Grass Technologies, West Warwick, RI, USA).

#### 2.4.4 Data acquisition

Data were recorded on a computer using an analog to digital converter (1401plus and Spike2 software, Cambridge Electronic Design, Cambridge, UK). EEGs and the EOGs were sampled at 250 Hz, the ECG at 500 Hz and the masseter EMG at 1000 Hz. GG EMG were sampled at 10 kHz and respiratory variables at 125 Hz.

### 2.5 Data analysis

In order to assess effects of alcohol on global genioglossal activity, a signal akin to a multi‐unit genioglossus EMG recording was created by summing all four genioglossal EMG channels during a 1‐minute section of the resting period at the start of the experiment (when the alcohol concentration in each participant was near its peak). Respiratory variables and genioglossal activity were calculated on a breath‐by‐breath basis and reported for all participants.

Individual MUs were identified and sorted in an identical fashion to our prior publications (Avraam et al., 2016). Briefly, each hypoxia trial was inspected by an experienced investigator (A.J. or J.T.) to determine whether at least one MU could potentially be identified and to ensure that there was no theta EEG activity or events such as body movements. If a minor body movement/swallow was identified during baseline or mid hypoxia, the trial was kept in the analysis but the individual breaths that were disrupted were not analysed. If, however, such events occurred at the end of hypoxia, during the hyperoxic breath or during recovery, the entire trial was excluded.

**FIGURE 2:**
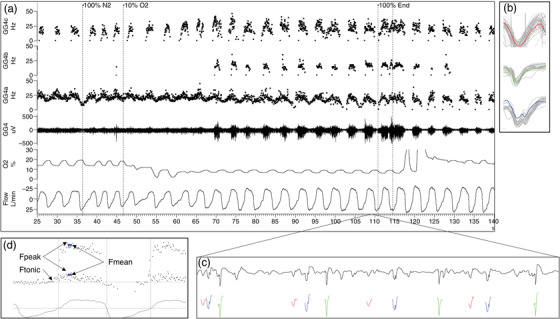
An illustrative example of the steps in sorting motor units. One hypoxia trial in an individual 22‐year‐old female participant studied following alcohol consumption (panel a). First, the Spike2 wavemark function was used to automatically identify three unique motor units whose unique spike profile (average of 40 motor units is seen in panel b) could be identified based on the amplitude and shape of each spike. Next, the individual wavemark (as visible in panel c) and firing frequency was inspected to correct any misclassified motor units, or add spikes when they occurred simultaneously or were missing. Finally, after the sorted motor units had been checked for accurate classification, the peak, mean and tonic firing frequencies were calculated for every breath across the trial (panel d).

Next, MU action potentials (spikes) were identified by Spike2 analysis software using a spike‐triggered threshold voltage (set individually for each EMG channel) and based on the amplitude, duration and shape of spikes. Additional software (Spike2 script courtesy of Neuroscience Research Australia, Sydney, NSW, Australia) was then used to allow investigators to inspect and, if necessary, edit each spike based on its amplitude, shape and frequency. Finally, the decomposition of each EMG for each trial was reviewed and approved or, if considered inaccurate, returned for further sorting or discarded. The steps in this process are outlined in Figure 2.

In addition to the quantification of MU discharge activity (see below), each MU was classified according to two properties: the *within‐breath* discharge pattern during the pre‐hypoxia baseline period and the *within‐trial* discharge pattern. The within‐breath discharge pattern refers to the variation in a unit's discharge rate as a function of the respiratory cycle. Five of the six within‐breath discharge patterns identified by Saboisky et al. (2006) were identified in the current study: IP, IT, EP, ET and TT. To distinguish between tonic units with respiratory modulation (IT and ET units) and tonic units with minimal respiratory modulation (TT units), the presence of respiratory modulation was assessed by cross correlating a unit's discharge rate (instantaneous frequency values) with tidal volume over a breath. Consistent with prior studies (Saboisky et al., 2006), units with average cross correlation values of less than 0.4 were designated as TT units. IP versus EP modulation was determined by visual inspection in relation to the respiratory cycle.

Within‐trial discharge pattern refers to modulation of MU activity over the course of a trial. Three within‐trial discharge patterns were identified: constant, recruited and de‐recruited. Constant units were those that were active throughout a trial from the beginning of baseline until the end of the normoxic period (brief periods of inactivity that occurred only during a movement or swallow late in the normoxic period did not affect/preclude this classification). Recruited units were units that were silent during baseline and became active subsequent to the onset of hypoxia. De‐recruited units were defined as units that were active during baseline but became inactive during the hypoxic or normoxic periods (this classification did not include units that became inactive only during brief body movements or swallows).

#### 2.5.1 Breath‐by‐breath measurements of MUs and respiratory activity

The discharge rate of each MU was quantified as in our prior studies (Avraam et al., 2021; Trinder et al., 2013) using software developed by Neuroscience Research Australia (Spike2 script) (Luu et al., 2020; Nguyen et al., 2019), which determines firing characteristics of each motor unit for every breath. Three measures were calculated for each breath across a trial: peak frequency (peak of the instantaneous frequency with a 200 ms running average for each breath); mean frequency (the mean of the instantaneous frequency averaged over 200 ms); and tonic frequency (the mean firing rate over 500 ms of the non‐respiratory modulated phase). In addition, minute ventilation (*V̇*_I_), peak inspiratory flow (PIF), tidal volume (*V*_t_), respiratory rate (RR), end tidal CO_2_ partial pressure ($P_{\mathrm{ETC}O_{2}}$), average inspiratory O_2_ concentration (mask O_2_) and nadir oxygen saturation ($S_{pO_{2}}$) were calculated for each breath by the script.

### 2.6 Statistical analyses

#### 2.6.1 Global genioglossus EMG activity and resting respiratory activity

The respiratory variables and the sum EMG values during 1 min of the resting period at the start of the experiment were compared between alcohol and placebo conditions using a paired Student's *t*‐test.

#### 2.6.2 Discharge patterns of MUs

The within‐breath distribution of discharge patterns (IP, IT, ET and TT units) and within‐trial distributions (Constant, Recruited and De‐recruited) were compared between conditions using chi‐square analysis.

#### 2.6.3 Quantification of MU activity

MU activity was quantified for four phases as follows: baseline (BL), defined as the average of the last three baseline breaths; hypoxia (HOX), defined as the average of the last three hypoxic breaths, hyperoxia (HYP), defined as the breath on which the 100% O_2_ reached the mask (first breath on which peak Mask O_2_ rose above 30%) and normoxia, defined as the value for each of the eight breaths (N1 to N8) following the hyperoxic breath and assessing the afterdischarge effect. Thus, the initial analysis of each variable consisted of a 2 (condition) × 11 (breath BL–N8) ANOVA. In order to assess both rate coding (firing frequency changes of active units) as well as influences of recruitment/de‐recruitment, two separate ANOVAs were performed as per prior studies (Avraam et al., 2021; Wilkinson et al., 2008). To assess rate coding (MU firing frequency changes), the data for any MU that did not fire for a particular breath were replaced with the mean firing rate of all units that were active on that breath. In order to assess recruitment/de‐recruitment, the data for any MU that did not fire for a particular breath was replaced with a zero. Due to the multiple (24) ANOVAs performed, *P* < 0.002 was considered statistically significant in ANOVA analyses. The α level was set at *P* < 0.05 for all other comparisons. When significant ANOVA effects were observed, *post hoc* analyses were conducted to investigate effects of hypoxia (BL compared to HOX) and/or afterdischarge (BL compared to breaths N1 through N8). *P* < 0.05 was considered significant for *post hoc* comparisons.

## 3 RESULTS

One participant vomited prior to the start of data collection in the alcohol condition and so all data from this participant were excluded, leaving 15 participants (7 men, 8 women) from whom data were recorded in both conditions. These participants were aged 23.8 ± 5.1 years and had a body mass index of 23.7 ± 3.5 kg/m^2^. At the start of the experiment, their BrAC was 0.07 ± 0.02% (range 0.052% to 0.099) for the alcohol condition and 0.00% for the placebo condition. Participants were able to correctly distinguish the alcohol from placebo condition.

For three participants no MUs were identified in the alcohol condition and for four participants no MUs were identified in the placebo condition. In order to retain a matched sample in each condition, MU data were only included in the nine participants who had MUs recorded in both conditions. These nine participants (5 women and 4 men) were aged 23.9 ± 5.1 years and had an average body mass index of 25.2 ± 3.2 kg/m^2^. A total of 96 hypoxia trials were performed in the placebo condition (range 9–12 per participant) with 94 trials performed in the alcohol condition (range 9–11 per participant). The BrAC was 0.00% for all measurements in the placebo condition whereas it was 0.06 ± 0.02% (range 0.052–0.072%) at the start of recording in the alcohol condition, declining to 0.04 ± 0.01% (range 0.032–0.055%) by the last hypoxic trial.

### 3.1 The effect of alcohol on genioglossal activity and respiratory parameters at rest

For the 15 participants with data available during the rest period before hypoxic episodes, global genioglossal activity did not differ between alcohol and placebo conditions (peak inspiratory EMG = 119.3 ± 44.1 vs. 126.5 ± 51.9 μV, respectively, *P* = 0.53; Expiratory tonic EMG = 77.2 ± 21.2 vs. 83.1 ± 24.3 μV, respectively, *P* = 0.32). Likewise, minute ventilation (placebo 8.3 ± 1.18, alcohol 8.3 ± 1.86 l/min, *P* = 0.9), breathing frequency (placebo = 15.4 ± 3.22, alcohol 15.4 ± 4.79 breaths/min, *P* = 0.9) and duty cycle (placebo = 0.35 ± 0.05, alcohol = 0.35 ± 0.05, *P* = 0.9) did not differ between conditions.

In order to quantify the effect of alcohol on MU activity, the 1 min baseline periods prior to hypoxia were examined throughout the entire protocol for the presence of MUs in the nine participants with MUs in both conditions. Sixty‐eight MUs were identified in the placebo condition whereas 67 were identified in the alcohol condition. The distribution of MUs (Table 1) differed significantly between conditions (χ^2^ = 10.72, *P* = 0.013) with fewer IP and IT units identified in the placebo condition than in the alcohol condition. However, neither the peak (placebo 21.2 ± 4.28, alcohol 22.4 ± 4.08 Hz, *P* = 0.089), nor mean (placebo 18.0 ± 4.05, alcohol 19.1 ± 3.6 Hz, *P* = 0.085) nor tonic (placebo 13.5 ± 6.93, alcohol 11.5 ± 6.99 Hz, *P* = 0.102) frequency of the MUs present differed between conditions.

**TABLE 1:** The number of MUs of each class recorded during the baseline periods for alcohol and placebo conditions.

	Placebo		Alcohol	
MU class	Number of participants	*n* (%)	Number of participants	*n* (%)
ET	8	29 (43)	6	22 (33)
IP	4	5 (7)	5	10 (15)
IT	4	8 (12)	4	20 (30)
TT	8	26 (38)	6	15 (22)
Total		68		67

The distribution of motor units differed between alcohol and placebo conditions according to the chi square test (χ^2^ = 10.715, *P* = 0.013). Abbreviations: ET, expiratory tonic; IP, inspiratory phasic; IT, inspiratory tonic; TT, tonic.

### 3.2 The effect of alcohol on respiratory and genioglossal afterdischarge

**FIGURE 3:**
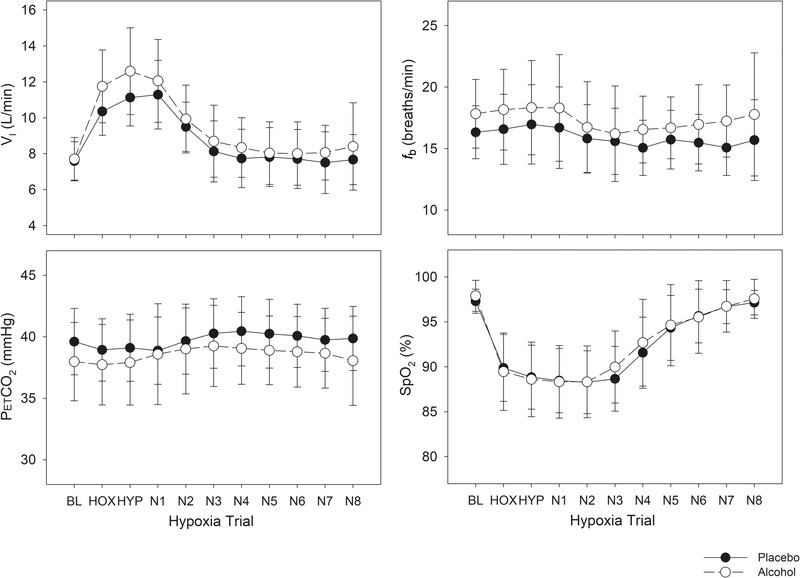
Changes in respiratory variables across baseline (BL), hypoxia (HOX), hyperoxia (HYP) and recovery breaths (N1–N8) in alcohol and placebo conditions in all nine participants. *f*_b_, breathing frequency; $P_{\mathrm{ETC}O_{2}}$, end tidal carbon dioxide levels; $S_{pO_{2}}$, arterial oxygen saturation; *V̇*_I_, inspired minute ventilation. Repeated measures ANOVA revealed significant differences existed across breaths for all four variables (*P* < 0.001), and the alcohol condition differed from the placebo condition for *V̇*_I_ (*P* = 0.009) and *f*_b_ (*P* = 0.007). Finally, end tidal CO_2_ differed between conditions over breaths (interaction effect *P* = 0.011).

In the placebo condition MUs were identified in 48 (50%) hypoxia trials whereas in the alcohol condition MUs were identified in 38 hypoxia trials (40%) (χ^2^ = 1.76, *P* = 0.185). Small but significant differences between the alcohol and placebo conditions were observed in some respiratory variables when the trials that resulted in MUs were assessed. Specifically, greater hyperventilation was observed in response to hypoxia in the alcohol condition (Figure 3a, ANOVA Breath *P* < 0.001, Condition *P* = 0.009 and Interaction *P* = 0.051), which was predominantly due to higher breathing frequency in the alcohol condition (Figure 3b, ANOVA Breath *P* < 0.001, Condition *P* = 0.007, and Interaction *P* = 0.398). $P_{\mathrm{ETC}O_{2}}$ also differed between conditions (Figure 3c, ANOVA Breath *P* < 0.001, Condition *P* = 0.055, and Interaction *P* = 0.011). These effects occurred even though there was no difference in oxygen saturation between conditions (Figure 3d, ANOVA Breath *P* < 0.001, Condition *P* = 0.627 and Interaction *P* = 0.364).

**FIGURE 4:**
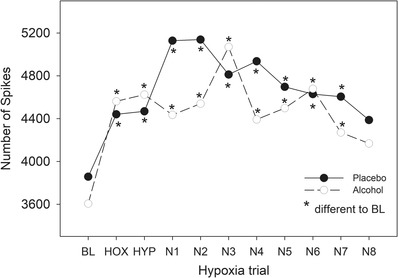
The total sum of single motor units (MUs) identified for all nine participants across all breaths of baseline (BL), hypoxia (HOX), hyperoxia (HYP) and recovery (N1–N8) in the alcohol and placebo conditions. Repeated measures ANOVA on the individual sum of motor units per breath revealed a significant effect of Breath (*P* < 0.001), but no significant Condition (*P* = 0.298) or Interaction (*P* = 0.155) effect. *Difference from BL.

During the hypoxia trials, 101 MUs were identified in the placebo condition whereas only 88 MUs were identified in the alcohol condition. The total number of MUs active over the protocol (an indication of overall muscle activity) did not differ between conditions (Figure 4, ANOVA, Breath *P* < 0.001, Condition *P* = 0.298, Interaction *P =* 0.155). The number of MUs was elevated above baseline until breath 7 of normoxia indicating afterdischarge occurred. Within‐breath discharge patterns as a function of within‐trial discharge patterns and condition are shown in Table 2. The proportion of IP and IT units that were constant over the hypoxia period was slightly higher in the alcohol condition, whereas slightly more MUs were recruited in the placebo condition. However, the total within‐breath distribution of all units did not differ between conditions (χ^2^ = 5.44, *P* = 0.14).

**TABLE 2:** The within‐breath discharge pattern of MUs according to different within‐trial patterns in alcohol and placebo conditions.

	Placebo				Alcohol			
	Constant	Recruited	De‐recruited	Total	Constant	Recruited	De‐recruited	Total
EP						1		1
ET	25 (71)	7 (20)	3 (9)	35	16 (50)	11 (34)	5 (15)	32
IP	2 (9)	18 (82)	2 (13)	22	8 (50)	7 (44)	1 (6)	16
IT	6 (43)	8 (57)		14	18 (82)	3 (14)	1 (4)	22
TT	20 (66)	4 (13)	6 (20)	30	15 (88)	2 (12)		17
Total	53 (52)	37 (37)	11 (11)	101	57 (65)	24 (27)	7 (8)	88

The values are *n* (%). No significant differences in distributions of units were identified by chi square analysis (χ^2^ = 5.44, *P* = 0.14). Abbreviations: EP, expiratory phasic; ET, expiratory tonic; IP, inspiratory phasic; IT, inspiratory tonic; TT, tonic.

### 3.3 Analyses of recruitment/de‐recruitment and rate coding during hypoxia and recovery

As described earlier, two analyses were performed to independently assess recruitment/de‐recruitment (breaths where a MU did not fire were replaced with zeros) and frequency changes irrespective of recruitment (known as rate coding: breaths where a MU did not fire was replaced with the average firing frequency of all other active units).

**FIGURE 5:**
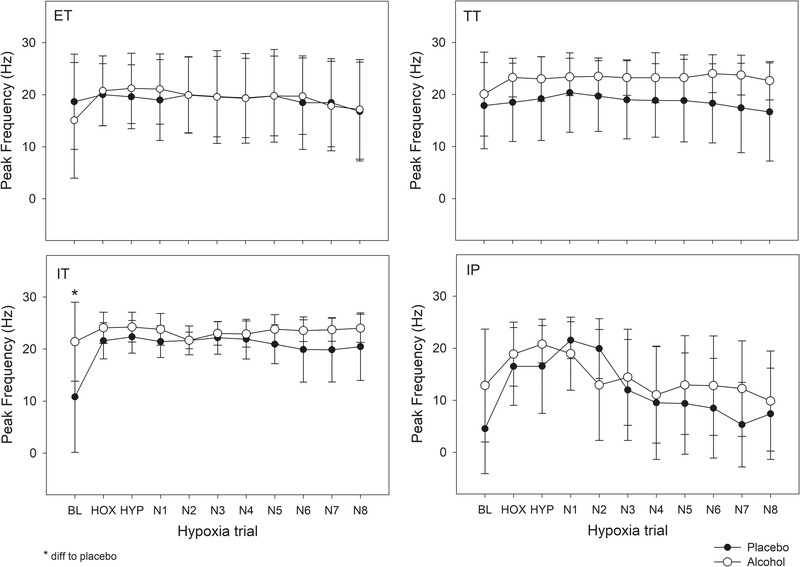
Peak firing frequencies of MUs across baseline (BL), hypoxia (HOX), hyperoxia (HYP) and recovery (N1–N8) breaths in alcohol and placebo conditions including zeros in the analysis when MUs did not fire in order to demonstrate recruitment of MUs. Repeated measures ANOVA indicated that recruitment of IT units was greater in the placebo versus alcohol condition (Interaction effect *P* = 0.001 as well as significant *P* < 0.001 breath effect) and IP units tended to differ (Interaction effect *P* = 0.002), but there were no statistically significant changes in ET and TT units over breaths (*P* = 0.04 and 0.113) or between alcohol and placebo conditions (*P* = 0.924 and 0.015). (Note: *P* < 0.002 was considered significant due to Bonferroni correction.) The number of participants (*N*) and number of units (*n*) for each unit type in the Placebo condition were IP *N* = 4, *n* = 22; IT *N* = 4, *n* = 14; TT *N* = 8, *n* = 30; ET *N* = 8, *n* = 35; and in the Alcohol condition were IP *N* = 5, *n* = 16; IT *N* = 4, *n* = 22; TT *N* = 6, *n* = 17; ET *N* = 6, *n* = 32.

The analysis including zeros indicated that no recruitment occurred for ET or TT units across hypoxia or recovery, nor did the mean or peak firing frequencies of these MUs differ between alcohol and placebo conditions (*P* = 0.004, *P* = 0.011, respectively). In contrast, however, there were significant changes in mean and peak firing frequencies of both IP and IT units across hypoxia (Breath effects, *P* < 0.001). The IT MUs also differed by alcohol condition (Figure 5, Interaction *P* = 0.001) whereas the change in IP units did not reach the Bonferroni corrected significance threshold (*P* = 0.002). Specifically, at baseline prior to hypoxia more IT units were active in the alcohol condition, but this difference disappeared during and after hypoxia. It is important to note that three participants contributed more than 10 MUs to a given unit type in a particular condition (IT alcohol, TT placebo and IP placebo conditions) potentially biasing the results. Therefore, data in Figure 5 were regenerated by first averaging MU frequency changes within participants, and the results were very similar (see Supporting information, Figure S1).

In the second analysis, no evidence of rate coding existed for ET, TT or IT units (no significant breath effect for mean (*P* = 0.042, *P* = 0.530 and *P* = 0.058, respectively) or peak (*P* = 0.065, *P* = 0.442 and *P* = 0.062, respectively) firing frequencies). Significant breath effects were present for IP mean (*P* < 0.001) and peak (*P* < 0.001) frequency (frequencies reduced over recovery breaths) but these did not differ by condition (*P* = 0.77 and 0.489 for peak and mean respectively).

## 4 DISCUSSION

The aim of this study was to assess the effect of alcohol on genioglossus muscle activity. Contrary to expectations, and two prior studies in humans (Krol et al., 1984; Leiter et al., 1987), only modest changes in muscle activity were observed following alcohol consumption. We hypothesized that inspiratory modulated (IP/IT) MUs would be reduced in the alcohol condition, whereas the activity of expiratory and tonic units would remain unchanged. In contrast, we observed slightly more inspiratory (IP/IT) MUs in the alcohol condition versus the placebo condition. It is noteworthy that the proportion of IP and IT MUs identified in both the placebo (13%) and alcohol (30%) conditions were lower in this study compared to prior studies where approximately 50% of MUs in the genioglossus have been reported to be of the IP/IT class (Nicholas et al., 2010; Saboisky et al., 2006; Wilkinson et al., 2008). This may in part be due to the fact that in 3 of 9 participants MU wire electrodes were placed more superficially (20 mm) than in the prior studies (25 mm depth). While this was done to ensure that EP and ET units were recorded in sufficient numbers to examine alcohol effects, it does make comparisons of MU proportions to prior studies more difficult. Importantly however, the differences in electrode placement do not explain the difference in IP/IT MUs between the alcohol and placebo conditions, because electrode depth was consistent within the nine individuals. A possible alternative explanation for the increase in IP/IT units during the alcohol condition is that alcohol caused increased nasal congestion (and therefore increased airway resistance), and as such there was a slight loading of the airway. Increased nasal and pharyngeal resistance has been observed following alcohol intake in some (Eckert et al., 2010; Robinson et al., 1985; Series et al., 1990) but not all (Dawson et al., 1997) prior studies. Oesophageal/epiglottic pressures were not measured in this study so it is not possible to know whether airway resistance differed between conditions. Duty cycle often changes with resistance during wakefulness, but was not altered in this study, against the idea that resistance was elevated in the alcohol condition.

The finding that activity of IP and IT units was not reduced following alcohol consumption may have implications for our model of genioglossus motor control outlined in Figure 1. It is possible that in humans alcohol does not alter the sleep–wake control centre's input to the hypoglossal motor nucleus as proposed in Figure 1 (red dashed box). This would be in contrast to the elegant rat study conducted by Vecchio et al. (2010) which showed that direct infusion of ethanol into the hypoglossal motor nucleus had minimal effects in anaesthetized rats, but that systemic administration of ethanol (intraperitoneal injection) reduced genioglossal activity during wakefulness only, suggesting that alcohol reduces wakefulness input to the hypoglossal motor unit. While species and methodological differences may account for the different findings between the current study and that by Vecchio et al. (2010), it is also possible that low baseline levels of genioglossal activity in our healthy young volunteers minimized the chances of observing effects of alcohol. Further work will be required to investigate these possibilities.

MU activity was also measured during hypoxia and recovery in order to assess whether genioglossal afterdischarge was altered by alcohol. Perhaps not surprisingly given the minimal differences between conditions at baseline, alcohol did not alter the number of MUs identified during hypoxia or the afterdischarge period. The duration of genioglossal afterdischarge was similar between the alcohol and placebo conditions. This occurred despite slight differences in respiratory variables, with slightly greater hyperventilation and greater breathing frequency changes during the alcohol than placebo condition, notwithstanding a comparable degree of desaturation between conditions. Prior studies in humans have generally reported unchanged hypoxic and hypercapnic ventilatory responses unless the alcohol dose is very high (Dawson et al., 1997) and so the reason for these differences is unclear. There were slight differences between conditions in the classes of MUs identified, with more IP and IT units being present at baseline in the alcohol condition and less recruitment of IT units during hypoxia and recovery as compared to the placebo condition.

### 4.1 Clinical implications

**FIGURE 6:**
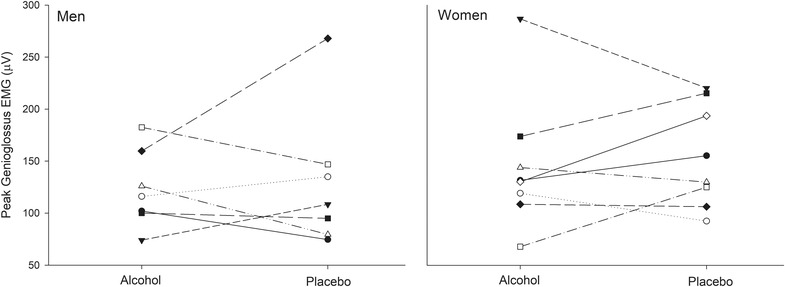
Peak inspiratory global genioglossus EMG (sum of all MU channels) in men (*n* = 7) and women (*n* = 8) at the beginning of the protocol in alcohol and placebo conditions. Each line represents data for an individual participant. There were no significant differences in muscle activity between alcohol and placebo conditions in either sex (Student's *t‐*test for alcohol vs. placebo, *P* = 0.75 in men and *P* = 0.57 in women).

Although this study was not designed to assess the effect of alcohol on overall genioglossal activity, the ‘global’ measure of genioglossal activity suggested that there were minimal changes in activity at a breath alcohol concentration of ∼0.07%. This was surprising given previous reports of reduced multi‐unit activity of genioglossus as a result of alcohol consumption (Krol et al., 1984; Leiter et al., 1987). The two prior reports used slightly higher doses of alcohol (blood alcohol concentration ∼0.08%) and studied participants who were similarly aged but in a semi‐recumbent position (as opposed to supine in the current study). The first study in six men and six women did find the depressant effect of alcohol was more pronounced in men than in women (Krol et al., 1984) and led to a subsequent study which demonstrated women had reduced genioglossal activity in response to alcohol only in the follicular menstrual phase (Leiter et al., 1987). Two women in the current study were on the oral contraceptive pill for both conditions and the menstrual phase was matched between conditions in three other women (both studies in the follicular phase for two participants and both in the luteal for one participant). In the remaining two women menstrual phase data were unavailable. However, in the current study, the global muscle activity measure did not appear to differ between sexes (individual data shown in Figure 6). Thus, it appears unlikely that sex or hormone differences explain the lack of effect of alcohol on global genioglossal activity observed in this study. Vecchio et al. (2010) proposed that the effect of alcohol may be more pronounced when baseline muscle activity is elevated, so it is possible that the relatively young age and low BMI of participants in our study contributed to low baseline genioglossal activity and therefore minimal alcohol effects. However, even when muscle activity was increased with hypoxia, alcohol effects were not observed. Further research is required to determine whether snorers or individuals with OSA have more marked genioglossus muscle activity changes with alcohol and if so, what units are modified by alcohol.

If future studies conducted during sleep are consistent with the current study and demonstrate only modest effects of alcohol on the activity of the genioglossus muscle, then the cause of the increase in snoring and OSA severity with alcohol intoxication would appear likely to be due to some other factor. As mentioned, one likely candidate is increased nasal resistance as a result of nasal congestion (Eckert et al., 2010; Robinson et al., 1985; Series et al., 1990). Another possible contributing factor is that the arousal threshold is altered by alcohol. Previous studies have reported longer apnoea durations (Berry et al., 1992; Issa & Sullivan, 1982) or more negative oesophageal/epiglottic pressures before arousal (Berry et al., 1992) following alcohol consumption, indicating that individuals were less easily awoken by respiratory stimuli when intoxicated. Further physiological studies are required to clarify the mechanism of alcohol‐induced worsening of OSA.

### 4.2 Limitations

Despite its strengths, the current study has some limitations. First, with respect to the clinical implications of this study, the fact that healthy participants were studied during wakefulness makes it difficult to extrapolate the findings to comment on the implications for sleep apnoea. Second, the number of participants contributing to some MUs was quite low (*n* = 4). As a result, we treated MUs as individual data points whereas they are not truly independent. However, the results were near identical when averaged within an individual first, suggesting that this did not alter our findings/conclusions. Third, the ability to identify MUs may have differed between conditions. Specifically, if alcohol reduced background EMG activity, the identification of MUs may have been easier in the alcohol condition. This could have contributed to the lack of reduction in the numbers/proportions of MUs observed. However, if this was the case we would expect all classes of MUs to be affected equally, and therefore not alter the distribution of MUs observed. Further, the global measure of genioglossal activity would be expected to have reduced activity in the alcohol condition, which was not observed.

### 4.3 Conclusion

This study conducted in healthy young men and women during wakefulness found that there was no reduction in genioglossal activity while participants were under the influence of alcohol at a breath concentration of ∼0.07%. There were slight differences in the firing patterns of MUs identified in the alcohol condition compared with placebo, with more inspiratory‐related units present. The duration of genioglossus afterdischarge was not altered when participants had consumed alcohol, but there did appear to be a slight reduction in recruitment of new IT units under the influence of alcohol. These findings raise questions regarding whether the increased severity of snoring and OSA observed after alcohol consumption are related to reduced genioglossus muscle activity or other factors.

## AUTHOR CONTRIBUTIONS

This work was performed at the Melbourne School of Psychological Sciences, University of Melbourne, Melbourne, Australia. Amy S. Jordan, Fergal J. O'Donoghue, and John Trinder conceived the study and conducted analyses; Joanne Avraam, Andrew Dawson, Christian L. Nicholas collected the data; Joanne Avraam, Andrew Dawson, John Trinder, Monika D. Fridgant, Feiven Lee Fan, Amanda Kay, Zi Yi Koay, Rachel Greig analysed single motor units; all authors contributed to interpretation; Amy S. Jordan drafted the manuscript; all authors (except John Trinder) read and provided a critique of the manuscript. All authors (except John Trinder, deceased) have read and approved the final version of this manuscript and agree to be accountable for all aspects of the work in ensuring that questions related to the accuracy or integrity of any part of the work are appropriately investigated and resolved. All persons designated as authors qualify for authorship, and all those who qualify for authorship are listed.

## CONFLICT OF INTEREST

Philips Respironics provided nasal mask for use in this study free of charge, but were not involved in study design, data collection or analysis. No other competing interests exist

## Supporting information

Statistical Summary Document

Figure S1. Figure 5 duplicated but averaging motor units within a participant (*n* = number of participants)

Raw data

## Data Availability

Due to the size of data files they are not published on a server. However, the data that support the findings of this study are available from the corresponding author upon request.
