# Supplementary material for: The influence of alcohol on genioglossus single motor units in men and women during wakefulness
Source: Exp Physiol. 2022 Dec 19;108(3):491–502. doi: 10.1113/EP090580 (PMC10103883; doi:10.1113/EP090580)
Supplement: Supplementary file 2 — Figure S1. Figure 5 duplicated but averaging motor units within a participant (n = number of participants) [file EPH-108-491-s001.pdf]

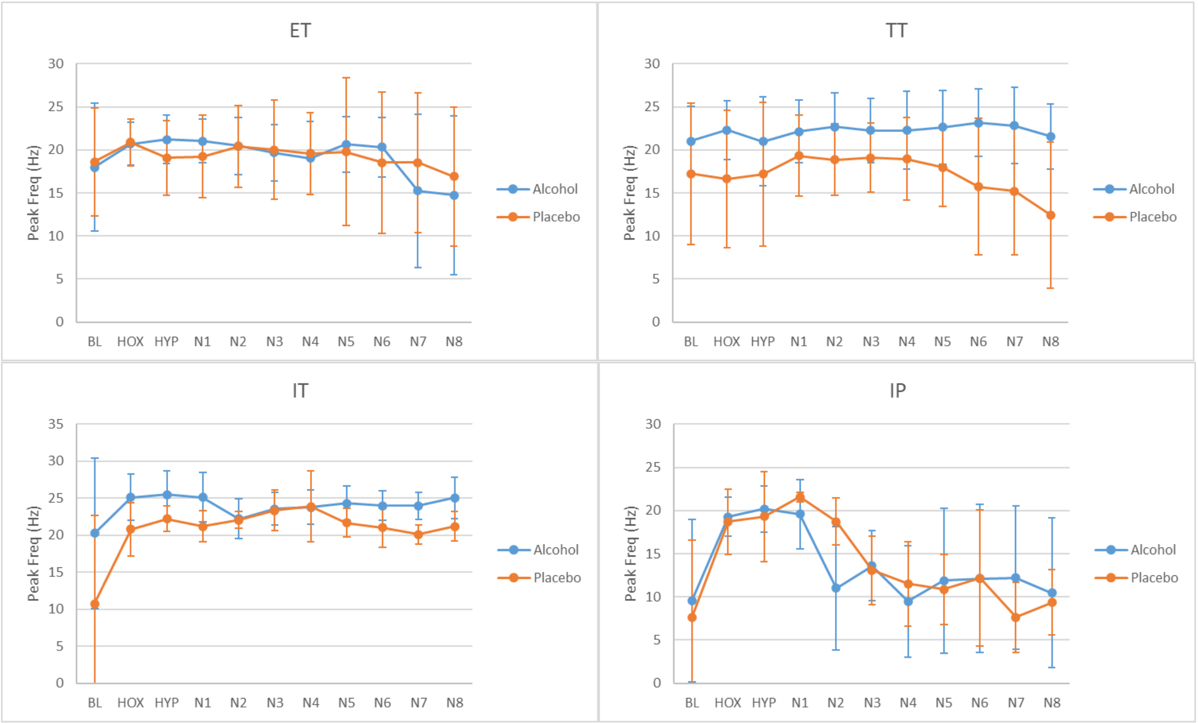

Supplementary Figure

Figure 5 reanalysed when averaged by the participant first. As can be seen the overall responses were consistent when averaging individual motor units within a subject before averaging participant responses. Thus we do not believe the problem of pseudo-replication likely contributed to the overall findings or interpretation.

Peak firing frequencies of MUs across baseline (BL), hypoxia (HOX), hyperoxia (HYP) and recovery (N1-N8) breaths in alcohol and placebo conditions including zeros in the analysis when MUs did not fire in order to demonstrate recruitment of MUs. The number of participants (N) for each unit type in the Placebo condition were IP N=4; IT N=4; TT N=8; ET N=8 and in the Alcohol condition were IP N=5; IT N=4; TT N=6; ET N=6.
